# The utility of a shortened palliative care screening tool to predict death within 12 months – a prospective observational study in two south African hospitals with a high HIV burden

**DOI:** 10.1186/s12904-019-0487-5

**Published:** 2019-11-13

**Authors:** Peter J. Raubenheimer, Cascia Day, Faried Abdullah, Katherine Manning, Clint Cupido, Jonny Peter

**Affiliations:** 1 Division of General Medicine, Department of Medicine, Groote Schuur Hospital and University of Cape Town, Cape Town, South Africa; 2 Division of General Medicine, Department of Medicine, Victoria Hospital Wynberg and University of Cape Town, Cape Town, South Africa; 3 Division of General Medicine, and Division of Immunology and Allergology, Department of Medicine, Groote Schuur Hospital and University of Cape Town, Cape Town, South Africa

**Keywords:** Palliative care, Prognosis, Hospitilization, Clinical decision-making

## Abstract

**Background:**

Timely identification of people who are at risk of dying is an important first component of end-of-life care. Clinicians often fail to identify such patients, thus trigger tools have been developed to assist in this process. We aimed to evaluate the performance of a identification tool (based on the Gold Standards Framework Prognostic Indicator Guidance) to predict death at 12 months in a population of hospitalised patients in South Africa.

**Methods:**

Patients admitted to the acute medical services in two public hospitals in Cape Town, South Africa were enrolled in a prospective observational study. Demographic data were collected from patients and patient notes. Patients were assessed within two days of admission by two trained clinicians who were not the primary care givers, using the identification tool. Outcome mortality data were obtained from patient folders, the hospital electronic patient management system and the Western Cape Provincial death registry which links a unique patient identification number with national death certificate records and system wide electronic records.

**Results:**

822 patients (median age of 52 years), admitted with a variety of medical conditions were assessed during their admission. 22% of the cohort were HIV-infected. 218 patients were identified using the screening tool as being in the last year of their lives. Mortality in this group was 56% at 12 months, compared with 7% for those not meeting any criteria. The specific indicator component of the tool performed best in predicting death in both HIV-infected and HIV-uninfected patients, with a sensitivity of 74% (68–81%), specificity of 85% (83–88%), a positive predictive value of 56% (49–63%) and a negative predictive value of 93% (91–95%). The hazard ratio of 12-month mortality for those identified vs not was 11.52 (7.87–16.9, *p* < 0.001).

**Conclusions:**

The identification tool is suitable for use in hospitals in low-middle income country setting that have both a high communicable and non-communicable disease burden amongst young patients, the majority under age 60.

## Background

Timely identification of people who are at risk of dying is an important first component of end-of-life care both in South Africa and Internationally [1–5]. Identification leads to earlier planning and initiation of palliative care. However, clinicians often fail to recognise that patients are in the final phases of their life, and have been reluctant then to discuss prognosis. This has resulted in the development of various trigger tools for patient identification that aim to predict prognosis more accurately and in turn introduce palliative care at an appropriate time [6, 7]. Such tools should ideally be evidence-based, transferable across healthcare systems, and should standardise the early identification process and thus may promote the systematic early identification of palliative patients.

Hospitalisation is an important medical encounter that increases the chance of identifying patients in the last year of life. Previous publications have shown that 12–35% of patients admitted to hospital will die within 12 months of admission [8–12]. Mortality risk assessment models for single diagnoses are often used by hospital specialists but are of limited value since most patients are admitted with advanced, long-term conditions which are multimorbid in nature [13]. Walsch et al. [14] reviewed the four widely used tools in General Practice; three of those, the Gold Standards Framework Prognostic Indicator Guidance (GSF-PIG) [15],the Supportive and Palliative Indicators Tool (SPICT) [16] and the Palliative Necessities CCOMS-ICO (NECPAL) [17] have been tested in hospital populations and have been shown to increase early identification of patients, potentially leading to more proactive care of patients who have palliative care needs. The GSF-PIG and NECPAL include the widely used ‘Surprise question’ (Would you be surprised if this patient died within the next 12 months?) and combined this with clinical indicators of advanced conditions.

Such tools have not been formally tested in South Africa (SA). The South African National Policy on Palliative care of 2017 suggested development of a SA Palliative Care assessment tool modelled on the GSF-PIG and SPICT, but also discusses that this tool still needs to be validated for general use [1]. A simplified version (in particular designed to fit on a single printed page) of the GSF-PIG has been used in two public sector hospitals in the Western Cape Province for several years already to aid clinicians in the identification and referral of patients for local palliative care programs [18].

This study aims to test the shortened GSF-PIG tool in patients admitted to general medical wards in two public sector hospital in South Africa, in the context of the double burden of disease [19].

## Methods

This study comprises a sub-study of a larger research project exploring the relationship between delirium and outcomes in a cohort of patients admitted to the acute general medical wards at two public hospitals in Cape Town (https://clinicaltrials.gov/ct2/show/NCT01916889). The two hospitals together admit acute general medical patients to approximately 220 beds and provide secondary and tertiary care for a local population of about 2 million people.

### Design and sampling

In this prospective, observational study, up to ten randomly selected patients of the daily acute medical intake on weekdays at each hospital were reviewed for study inclusion by a team of two trained clinicians at each hospital during consecutive 3 month periods (during Nov-Jan in one and during Feb–Apr in the 2nd hospital. All patients aged > = 18 and admitted for the first time in this time period as an acute, non-elective admission to the general medical wards could be included. Those refusing consent, not available in the ward on the day of sampling or having died before the day of sampling, were excluded.

### Procedure

Patients and patient notes were assessed within two days of admission by two trained clinicians who were not the primary care givers, using the identification sheet in ***Additional file*** ***1*** – a simplification of the Gold Standards Framework Prognostic Indicator Guidance (GSF-PIG) that has been used in these hospitals for two years as a clinician’s aid and referral tool. The tool includes the “Surprise Question” (SQ) – “would you be surprised if the patient were to die in the next 12 months”, and specific indicators of disease. The GSF-PIG general indicators were not included in the tool locally mainly to simplify the tool for use as a single one page referral tool. The tool allowed for an unspecified category (“Other”) in the indicators section, where the assessor would complete the reason/disease why death was expected. Traditionally, either a tick in the SQ box “Yes” or/and a tick in an indicator box would be considered to be a positive “identification” of a patient. Additional demographic data was collected from the patient, caregivers, electronic medical records and dispensing records, including previous diagnoses, previous medication history and a baseline Barthel Score. Investigations, including HIV screening and testing, were performed at the discretion of the attending physicians and with patient consent; an HIV-infected status was defined as any record of a positive HIV test (on formal ELISA) at any point prior to or during index admission dating back to January 2005 (the earliest available information on the National Health Laboratory results electronic system); an HIV-uninfected status as a formal negative laboratory ELISA during this admission or the 2 months preceding it; other patients were categorised as “HIV-unknown”. The electronic discharge letter was collected with final diagnosis based on ICD-10 coded primary diagnosis. Outcome mortality data were obtained from patient folders, the hospital electronic patient management system and the Western Cape Provincial death registry which links a unique patient identification number with national death certificate records and system wide electronic records. Patients for whom there were no reported mortality outcomes at the time of data extraction were assumed to have been alive at the end of the study.

### Data analysis

Data were analysed in Stata 14·2 (Stata Corp., College Station, TX) and GraphPad Prism 8.02 (GraphPad Software Inc., California) for Kaplan Meier Graphs. Continuous data was summarized either as means and standard deviations or medians and interquartile ranges, and count data was summarized as frequency and percent. Socio-demographic, clinical characteristics and outcomes were assessed for differences between HIV-uninfected, and HIV-infected or HIV-unknown patients. Associations between categorical variables were analysed using the chi-square test and Fisher’s exact, as appropriate. Wilcoxon rank-sum or Kruskal-Wallis test was used to compare continuous variables between two and three groups respectively.

Sensitivity, specificity, positive (PPV) and negative predictive value (NPV), likelihood ratios for the “identification tool” and its components (the “Surprise Question” and specific indicators) were calculated for both the cohort as a whole and in HIV-infected vs. HIV-uninfected patients. We also analysed the performance of the three individual components of the screening tool: the SQ alone, the SQ combined with the indicators and the clinical indicators alone. These tests were compared with McNemar’s test, and taking into account multiple comparisons, significant differences were defined a priori as *p* < 0.001. Kaplan Meier graphs for patients “identified” vs “non-identified” were drawn, and compared with the Log-rank test.

### Ethical approval

The study was approved by the University of Cape Town – Groote Schuur Hospital Human Research Ethics Committee (HREC 532/2017).

## Results

### Baseline characteristics of the study population (Table 1)

One thousand one hundred and six patients were randomised from the intake for potential enrolment into the study, of which 121 did not meet the inclusion criteria (73 were readmissions, 6 were under the age of 18, 8 were elective admissions and 34 were not admitted to the general medical wards). Another 163 were excluded (77 were not included in the data collection of the original main study due to its exclusion criteria of “coma” or “aphasia”, 22 had other incomplete data, 28 refused consent, 32 were not on the ward on the day of study and 4 died before testing) (***Additional file*** ***2***. STARD Flow diagram). Of the 822 patients included in the analysis the median age was 52 (37–67) and 46% were male. The baseline demographics are shown in *Table* *1*. Patients were admitted with a wide variety of diagnoses, of which the most common primary diagnoses were infection other than tuberculosis (19%), tuberculosis (12%), acute coronary syndrome (12%), stroke (8%), heart failure (8%), exacerbation of chronic obstructive airways disease (5%) and cancer (4%). Multi-morbidity was very common – 72% of patients had a previous chronic disease diagnosis requiring chronic medication before admission; 40% had 2 or more pre-existing chronic diseases. Patients were generally independent before admission; 76% had a Barthel Index of 100 and only 9% of patients had a pre-admission Barthel Index score of 50 or less.

**Table 1:** Baseline, diagnosis and outcomes overall and according to HIV status

	All		HIV-uninfected		HIV-unknown		HIV-infected	
	N (822)	(%)	N (440)	(53.50%)	N (204)	(24.80%)	N (178)	(21.70%)
Gender, *n* (%)								
Male,	378	(46.0)	234	(53.2)	70	(38.9)*	66	(37.1)*
Female	444	(54.0)	206	(46.8)	110	(61.1)	112	(62.9)
Age								
Median (IQR)	52	(37–67)	50	(39–62)	71	(60–78)*	35	(30–44)*
Primary diagnosis								
Infection (other than TB)	145	(19)	55	(13.3)	26	(16.0)	54	((31.6)*
Tuberculosis	92	(12)	32	(7.7)	4	(2.5)*	56	(32.7)*
Acute coronary syndrome	93	(12)	50	(12.1)	40	(24.7)*	2	(1.2)*
Stroke	66	(8)	28	(9.2)	28	(17.3)*	6	(3.5)*
CCF	60	(8)	38	(6.8)	8	(4.9)	6	(3.5)*
COPD	38	(5)	28	(6.8)	8	(4.9)	2	(1.2)*
Cancer	28	(4)	18	(4.3)	4	(2.5)	6	(3.5)
Barthell pre-admission function, *n* (%)								
< 50	72	(8.8)	26	(5.9)	24	(11.8)*	14	(7.9)
Mortality								
In-Patient	42	(5.1)	18	(4.1)	12	(6)	12	(6.7)
3mo	122	(14.8)	54	(12.3)	38	(19)	22	(12.4)
12mo	164	(20.0)	76	(17.3)	38	(19)	38	(21.3)

*p < 0.01 vs the HIV-uninfected group

*TB* Tuberculosis, *CCF* Congestive cardiac failure, *COPD* Chronic obstructive pulmonary disease

One hundred and seventy eight (22%) of the patients were HIV-infected and 440 (54%) confirmed HIV-uninfected. In 204 (25%) patients HIV status was not known. HIV-infected patients were more likely than HIV-uninfected patients to be female and were younger (median age 35; 30–44). Amongst HIV-infected patients, the most common admission diagnoses were communicable diseases (tuberculosis and infection), with non-communicable diseases being far less common. Patients not tested for HIV were much older (median age 71; 60–78) and the disease profile was similar to HIV-uninfected patients, with a larger proportion being admitted for non-communicable diseases, particularly stroke.

Overall inpatient mortality was 5.1%, 3-month mortality 14.8% and total 12-month mortality 20.0%. 12-month mortality in HIV-infected patients was 21.3 and 17.3% in HIV-uninfected patients with a non-significant OR for 12-month mortality in HIV-infected patients of 1.30 (0.87–2.0).

### Performance of the identification tool

The performance of the identification tool in predicting mortality at 12 months is shown in Table 2 for the population as a whole and divided by HIV-infected and -uninfected populations. In 144 patients identified by the SQ *only* (in whom no specific indicator box was ticked) the test had a good sensitivity of 89% (68–100%), but a very poor specificity of 8% (3–11%) and we did not analyse data using the SQ alone any further, but only the combination (of the SQ *and* the clinical indicators, as opposed to using the GSF-PIG Guidance of “***or***”) and an assessment based on only the specific indicators without including those only identified by the Surprise Question.

**Table 2:** Performance of the tests in the overall cohort (N 822)

	No IDed (%)	Sensitivity	Specificity	PPV	NPV	LR +	LR-
Overall							
SQ + criteria	366 (45%)	0.77 (0.71–0.83)	0.64 (0.60–0.68)	0.35 (0.30–0.40)	0.92 (0.89–0.94)	2.13 (1.87–2.43)	0.36 (0.27–0.48)
Criteria only	218 (27%)	0.74 (0.68–0.81)	0.85 (|0.83–0.88)*	0.56 (0.49–0.63)*	0.93 (0.91–0.95)	5.10 (4.15–6.26)*	0.30 (0.23–0.39)
HIV-uninfected							
SQ + criteria	166 (38%)	0.68 (0.58–0.79)	0.69 (0.64–0.73)	0.31 (0.24–0.38)	0.31 (0.24–0.38)	2.18 (1.76–2.71)	0.46 (0.33–0.64)
Criteria only	104 (24%)	0.71 (0.61–0.81)	0.86 (0.86–0.83)*	0.52 (0.42–0.62)*	0.52 (0.42–0.62)*	5.17 (3.85–6.95)*	0.34 (0.24–0.48)
HIV-infected							
SQ + criteria	76 (43%)	0.74 (0.60–0.88)	0.66 (0.58–0.74)	0.37 (0.26–0.48)	0.90()0.84–0.96)	2.15 (1.60–2.89)	0.40 (0.23–0.69)
Criteria only	34 (19%)	0.58 (0.42–0.74)	0.91 (0.87–0.96)**	0.65 (0.49–0.81)*	0.89 (0.84–0.94)	6.75 (3.69–12.37)*	0.46 (0.32–0.67)

*Ppv Positive predictive value, NPV - = negative predictive value, LR + = positive likelihood ratio; LR Negative likelihood ratio, SQ Surprise question*

*p < 0.001 for criteria only vs *SQ + criteria*

The combination of the SQ **AND** Clinical Indicators performed well; however when the indicators ***ALONE*** were used to predict outcome, without including the SQ, the best test performance was obtained with a similar sensitivity of 74% (71–83%) to the SQ and indicators combined, but an improved specificity of 85% (83–88%), a PPV of 56% (49–63%) and a negative predictive value (NPV) of 93% (91–95%). The indicator-only method of predicting outcome performed equally well in the HIV-infected cohort of patients as compared with the overall or the HIV-uninfected cohort. In HIV-infected patients sensitivity, PPV and Positive Likelihood ratio were better using the indicator alone vs indicator and SQ.

### Characteristics and outcomes of patients “identified” vs “non-identified”

Using the indicator-only component of the identification tool, 218 of the 822 patients were “identified” (IDed) as being in the last year of their life (Table 3). There were no gender differences between “identified” and “non-identified patients” (non-IDed), though identified patients were older (median age 61 vs 49). A greater majority of patients “identified” presented with stroke, heart failure, COPD and cancer; less with an infection or tuberculosis. Less of the identified patients were HIV-infected, but more were HIV-unknown. The in-patient, 3-month and 12-month mortality for the “identified” vs the “non-identified” patients were 16% vs 1.3, 48% vs 3 and 56% vs 7% respectively.

**Table 3:** Demographics and Outcome of “Non-identified and “identified” patients (Criteria only)

	“Non-identified”	“Identified”
	N (604)	N (218)
Gender		
Male	280 (46%)	98 (45%)
Female	324 (54%)	120 (55%)
Age		
Median (IQR)	49 (35–61)	61 (48–75)
lowest - 30	100	14
31–40	80	14
41–50	104	20
51–60	112	24
61–70	56	44
71–80	60	48
81 and older	22	28
Primary diagnosis		
Infection (other than Tb)	123 (20.4%)	22 (10.1%)
Tuberculosis	78 (12.9%)	14 (6.4%)
Acute coronary syndrome	85 (14.1%)	8 (3.7%)
Stroke	26 (4.3%)	40 (18.3%)
CCF	22 (3.6%)	38 (17.4%)
COPD	18 (3.0%)	20 (9.2%)
Cancer	10 (1.7%)	18 (8.3%)
HIV status		
Uninfected	336 (55.6%)	104 (47.7%)
Infected	144 (23.8%)	34 (15.6%)
Unknown	114 (18.9%)	66 (30.3%)
Refused	10 (1.7%)	14 (6.4%)
Barthell pre-admission function		
< 50	52 (8.6%)	20 (9.2%)
Mortality		
In-Patient	8 (1.3%)	34 (16%)
3mo	18 (3.0%)	104 (48%)
12mo	42 (7.0%)	122 (56%)

**Fig. 1:**
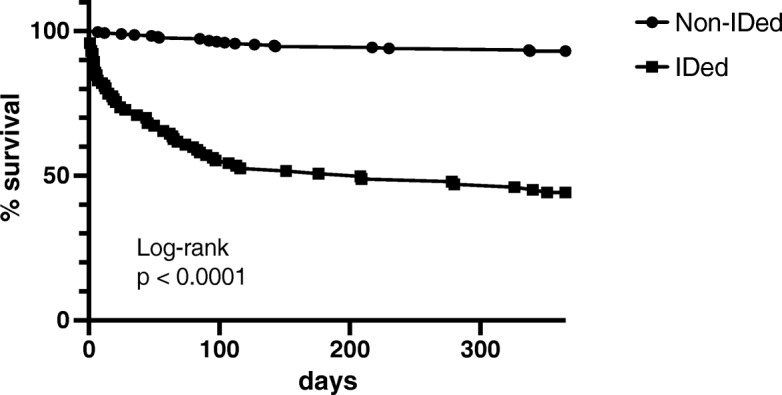
Kaplan Meier Survival curves for the “Identified” vs “Non-Identified” patients

Figure 1 shows the Kaplan Meier survival curve for the IDed vs non-ID patients. Survival was significantly worse for the “IDed” patients (*p* < 0.0001) with a hazard ratio for 12-month mortality for the IDed versus the non-IDed of 11.52 (7.87–16.9; *p* < 0.001). The tool strongly predicted mortality particularly in the next 3 months: 104 of 122 IDed patients (85%) vs 18 of 604 (3%) non-IDed patients died by 3 months after admission.

**Fig. 2:**
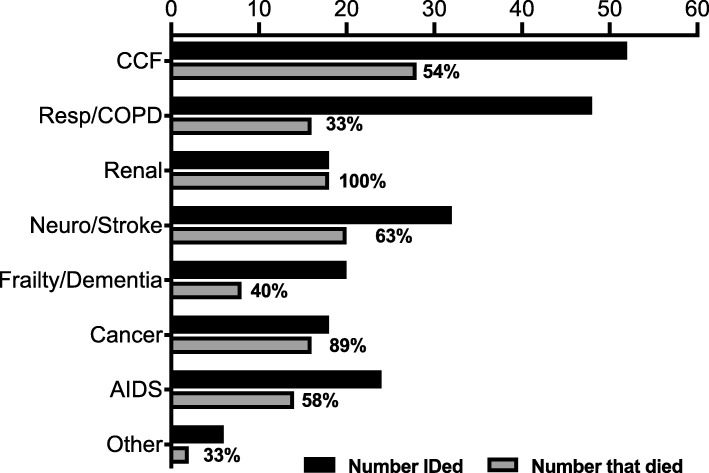
Number of patients “identified” by criteria and number that died at 12 months

Figure 2 shows the total number of patients identified according to the specific indicator met, and the number and percentage that actually had died at 12 months. Patients were identified from all the indicators, but heart failure and respiratory disease were the most common, with neurological disease (predominantly stroke) with the second highest frequency. The main indicator that was not specified and where the assessor completed the “other” box was liver failure. 12-month mortality was best predicted for patients with renal failure (100% correct), cancer (90% correct) and worst for patients with respiratory/COPD (33%), dementia/frailty (40%) and liver failure (33%). Fifty four percent of patients that fulfilled the heart failure criteria and 58% of patients that fulfilled the AIDS criteria had died by 12 months.

## Discussion

### Main findings

In this cohort of patients admitted to acute medical wards in a country with a high burden of communicable and non-communicable disease and a high prevalence of HIV, a simple checklist of specific indicators (based on the GSF-PIG) was able to identify patients with nearly 12-fold increase in hazard of death within 12-month of admission. Diagnostic accuracy measures showed good sensitivity (74% (71–83%)) and specificity (85% (83–88%)), a low to moderate positive predictive value (56% (49–63%)) and high negative predictive value (93% (91–95%)). There was no significant advantage to adding the “Surprise Question” to the specific indicators to identify such patients in this study.

The high burden of non-communicable diseases reflect the mortality statistics of South Africa [20], and the young age of patients dying are typical of developing low and middle income economies. The prediction tool’s indicators performed equally well in a younger cohort of HIV-infected patients as in an older cohort of HIV-uninfected patients. Using this tool clinicians were better able to identify patients in the last year of life with renal failure (100% IDed) and cancer (90% IDed) than with heart failure (54% IDed) or respiratory disease (33% IDed). Other studies have previously shown that predicting mortality in organ specific disease such as heart failure or respiratory failure is more difficult for clinicians than for cancer [21]. The accurate prediction of death of patients with renal failure reflects the access to management in this hospital context – where acute dialysis is available, but chronic dialysis access is restricted [22], such that patients who died were likely largely excluded from chronic dialysis programmes..

### Strengths and limitations of this study

This study is the first study evaluating the utility of an identification tool for predicting which patients may be in the last year of their life, in a low to middle income country with a high HIV burden. The use of record linkage ensured that outcome could be assessed objectively. The prospective enrolment of a large number of patients from the acute medical services across two hospitals, a tertiary and a large general hospital are typical of the types of patients admitted nationally and these findings are likely generalisable across South and Southern Africa and may be very similar in other low or middle income countries with a similar burden of disease. Further generalisability was ensured by patients being assessed by generalists, not palliative care specialists.

Limitations of the study were that it was only performed in acute medical admissions where patients are acutely unwell and may not be relevant to the primary care outpatient setting. Patients were also assessed within two days of their admission, when all information may not have been available to make an accurate assessment. Mortality as reported should be considered a minimum: vital status was not obtained for all patients; patients who have moved to another Province would not have been captured as deceased. Futhermore it should be considered that this tool does not necessarily predict overall palliative care need.

### Comparison with the other studies

O’Callahan et al. [23] performed a similar evaluation of the (full) GSF-PIG tool in 501 patients admitted to a New Zealand teaching hospital, where the average age of patients admitted was 70 years, the major diagnosis was cancer and the 12-month mortality was 67.7%. In that setting the tool performed well and very similar to in our setting with a sensitivity of 62.6%, a specificity of 91.9 a PPV 67.6% of and a NPV of 90.0%. Another widely used prediction tool, The Supportive and Palliative Indicators Tool (SPICT) was validated on a cohort of 130 patients admitted acutely with organ failure to specialist beds. 48% of identified patients had died at 12 months, reasonably similar to our data here [16]. De Bock et al. [24] found a sensitivity of 84.1% and a specificity of 57.9% for SPICT in a retrospective cohort study in a geriatric population general and clinical indicators performed equally in that study. The predictive value of the NECPAL CCOMS_ICO© tool was evaluated in a prospective, longitudinal study in primary care centres and a hospital in Spain in 1057 patients, with a mean age of 81 years and a 12-month mortality of 27.0%. The sensitivity was 91.3%, specificity 32.9%, PPV 33.5 and NPV 91.0 [17]. The high number of false positives in comparison to our study may reflect the different nature of the patients or the tool itself (which includes the SQ, disease indicators but also general indictors of severity, disease progression, co-morbidity and resource usage).

### Implications of this research

There is a need to improve the quality of care for patients near the end of their life admitted to hospitals, and the recognition that a large number of patients admitted are in the final year of their life, and that clinicians have difficulty identifying such patients, have led to the development of simple “identification” tools to assist. Ideally such tools, if used in busy acute admission wards, need to be quick, simple to use and accurate, so that resources are allocated only as appropriate. This is even more relevant in low and middle income countries where the patient burden far exceeds the availability of clinicians. The simple one page tool is an example of how that may be possible. Importantly, inclusion of the Surprise Question offered no additional benefit above criteria specific indicators. The National Policy on Palliative Care for South Africa currently references both the SPICT and the GSF-PIG as potential tools for prognostication, It also suggests that a South African Palliative Care Needs Assessment Tool (SAPCNAT) is developed using a combination of criteria from both of the above-mentioned models [1]. This abbreviated GSF-PIG tool presented here is a practical alternative until a national validated tool is developed.

## Conclusions

This study is the first to evaluate a prediction tool (here a simplification of the GSF-PIG) as a prognostic screening tool in a middle income country. Used by generalists in patients admitted acutely to medical wards, the tool identified a subpopulation of 26% of inpatients at high risk of death, of whom almost two thirds died within a year. Additional studies in other populations and in other countries are needed to determine how this tool will perform, and to determine whether this tool leads to improved outcomes for patients. Future developments should address improved identification in patients with organ failure and those with HIV, and to formally evaluate the performance of general features of declining health as included in GSF-PIG and SPICT to see whether they provide incremental benefit. Future tools developed for similar populations should ideally be compared directly to this version.

## Supplementary information

**Additional file 1.** Identification tool.The Palliative Care Identification Tool used in the Study.

**Additional file 2.** STARD flow diagram.STARD flow diagram for assessing the screening tool to predict 12-month mortality on 822 patients.

## Data Availability

The corresponding author may be contacted regarding access to data.

## Abbreviations

HIV: human immunodeficiency virus.
GSF-PIG: Gold Standards Framework Prognostic Indicator Guidance (now called Proactive Identification Guidance)
Palliative Necessities CCOMS-ICO (NECPAL): In Spanish: NECesidades PALiativas Centro Colaborador de la Organización Mundial de la Salud – Institut Català d’Oncologia; in English: Palliative Needs World Health Organization Collaborating Centre – Catalan Institute of Oncology) (NECPAL tool)
NPV: Negative predictive value
ICD-10: International Classification of Diseases 10th Revision
ELISA: *enzyme-linked immunosorbent assay*
*COPD*: *Chronic obstructive pulmonary disease*
PPV: Positive predictive value
SA: South Africa
SPICT: Supportive and Palliative Care Indicators Tool
SQ: The “Surprise Question”: “Would you be susprised if this patient died in the next 12 months?”
