# Supplementary material for: The utility of a shortened palliative care screening tool to predict death within 12 months – a prospective observational study in two south African hospitals with a high HIV burden
Source: BMC Palliat Care. 2019 Nov 13;18:101. doi: 10.1186/s12904-019-0487-5 (PMC6854790; doi:10.1186/s12904-019-0487-5)
Supplement: Supplementary file 1 — Additional file 1. Identification tool.The Palliative Care Identification Tool used in the Study. [file 12904_2019_487_MOESM1_ESM.docx]

**Additional File 1.** Identification tool

| **Palliative Care IDENTIFICATION TOOL** | | | |
| --- | --- | --- | --- |
| Would you be surprised if the patient were to die in the next year? | | **Yes** | **No** |
| Does the patient fulfil any of the following criteria? | | | |
| **Condition** | **Criteria** | **TICK** | |
| **Congestive Cardiac Failure** | - Symptoms despite maximal medical therapy - Disabling Shortness of breath at rest (NYHA Class IV) - ≥ 5 Admissions in past 6 months - Other associated organ involvement |  | |
| **Respiratory/COPD** | - Disabling Shortness of breath at rest (NYHA Class IV) - ≥ 5 Admissions in past 6 months |  | |
| **Renal Failure** | - End stage renal disease (GFR <15ml/min) - Not suitable / Declined for dialysis |  | |
| **Neurological Disease/Stroke** | - Severely disabling - Progressive functional decline - Severe dysphagia - Recurrent fever and sepsis |  | |
| **Frailty / Dementia** | - Significant functional impairment - Unable to do ADLs - Incontinence - Recurrent infections |  | |
| **Cancer** | - Stage IV malignancy (Metastatic) - Not for (further) definitive treatment - Spends >50% of time in bed / bedridden |  | |
| **AIDS** | - Stage 3 or 4 disease with dementia - Severe cachexia - Neoplasm, Failure of HAART |  | |
| **Other** | - Specify: |  | |
