# Supplementary material for: The utility of a shortened palliative care screening tool to predict death within 12 months – a prospective observational study in two south African hospitals with a high HIV burden
Source: BMC Palliat Care. 2019 Nov 13;18:101. doi: 10.1186/s12904-019-0487-5 (PMC6854790; doi:10.1186/s12904-019-0487-5)
Supplement: Supplementary file 2 — Additional file 2. STARD flow diagram.STARD flow diagram for assessing the screening tool to predict 12-month mortality on 822 patients. [file 12904_2019_487_MOESM2_ESM.docx]

**Additional File 2.** STARD flow diagram for assessing the screening tool to predict

12-month mortality on 822 patients.

**Excluded**

**n=84**

- Deceased prior to test (n=4)
- Not in ward on day (n=32)

Potentially Eligible patients

n=985

**No index test**

**n=50**

- Could not obtain consent (n=28)
- Incomplete CRF (n=99)
  - Excluded in main study for coma or aphasia (77)
  - Other missing data (22)

Eligible patients

n=949

# Index test

# (Modified GSF-PIG)

n=822

Index test positive (“Identified”)

n =218

n=24

Index test negative

(“Not-identified”)

n =604

n=5

## Mortality database review

n=218

## Mortality database review

## n=604

Confirmed dead 42

Confirmed alive 36

Unsure (Assumed alive) 526

Confirmed dead 122

Confirmed alive 30

Unsure (assumed alive) 66
